# Forecasting Model: The Case of the Pharmaceutical Retail

**DOI:** 10.3389/fmed.2022.582186

**Published:** 2022-08-03

**Authors:** Aurelija Burinskiene

**Affiliations:** Department of Business Technology and Entrepreneurship, Business Management Faculty, Vilnius Gediminas Technical University, Vilnius, Lithuania

**Keywords:** forecasting, model, drug, accuracy, shortage, planning levels

## Abstract

**Introduction:**

Many forecasting methods are used to predict sales, such as the moving average method, naive method, exponential smoothing methods, Holt's linear method, and others. The results brought by these models are quite different. Forecast delivered by the naive method is entirely accurate for an extended period, like 3–5 years, Holt's methods are bringing accurate one-year period forecasts. The planning decisions have several levels, meaning different forecasting results. However, the authors that are testing various forecasting methods are not discussing results researched in different planning levels (retail chain and different pharmacies). The study is given to the construction of the forecasting model covering both planning levels, which later is empirically tested for the Lithuania retail case:

**Purpose:**

The development of the forecasting model for reduction of shortages in drug supply. To achieve this goal, the author revises the improvement of drug availability weekly.

**Research Methodology:**

The construction of the forecasting model is incorporating outliers' detection methods and sales by pharmacies to minimize shortage. In the forecasting model, the author uses Theil's U_2_ test to evaluate forecasting accuracy.

**Findings:**

During analysis, the author constructs the model application for forecasting drug sales where weekly availability is highly recommended. The results show that forecasting on individual pharmacies level using the integration of these plans approach leads to higher accuracy.

**Research Limitations:**

The research covers 3 months of sales data. Das and Chaudhury suggest for short-sales period products use 36 days' time horizon. Ayati et al. discuss short and long-term time horizons for planning sales of drugs. Kanyalkar and Adil analyzed multi-site production and suggest that the time horizon should cover the longest lead time required for delivery of raw material, which is 12 weeks, and select 3 months (i.e., 13 weeks) as short-term time period horizon. Wongsunopparat and Chaveesuk forecast drug sales for 1-month and 12-month periods and compare the results. In this study, the focus is on short-term time-horizon, which is considered as 3 months period and also represents the longest lead-time. In the future, the study could review other periods. The author has incorporated the review of eight forecasting methods into the study by leaving other forecasting methods unresearched. Future studies could also incorporate different ARIMA methods into shortage reduction case analysis.

**Practical Implications:**

Presented forecasting model could be useful for practitioners, which analyze the reduction of the shortage of prescribed drugs. There the revision of repeated purchases is recommended for national authorities, wholesalers, and pharmacies aiming to minimize shortage.

**Originality/Value:**

The analysis to reach the highest forecast accuracy and identification of a forecasting approach which responds to the fluctuation of weekly sales for the whole pharmacy chain and separate pharmacies. The study contributes to drug sales review, where most authors analyze the total volume, which is not separated by pharmacies.

## Introduction

The supply chain of pharmacies often deals with forecasting questions. The construction of the forecasting model (1), which covers the sales of pharmacies and incorporates the drug supply aspects, and minimizes drug shortage cases is essential to the research question. In other words, forecasting sales helps to plan drugs' supply and respond to actual patients' demand which has weekly patterns (2). Drug shortage could be differed by medicine access level, retail and hospital-only. Ayati et al. (3) notify that the induced demand for stocking medication, which is, may cause periodic shortages in the pharmacies. Among the forecasting techniques, researchers are dealing with naive forecasting, average moving (4), and more sophisticated methods such as exponential smoothing and others.

In general, one of ten products is not available in pharmacy due to a shortage in supply. The occurrence of shortage disbalances supply and its recovery period is too long (5). Usually, pharmacies do not record count missed sales when forecasting (6). The author builds the forecasting model by incorporating missing data and extraordinary cases to it to get proper sales data for planning supply.

To improve supply, the author proposes using the prediction of average sales per week, which helps to protect the data from being downwards or upwards. To avoid the events of shortage and to shorten the recovery period, missing sales data must be set up. For high uncertain cases, the author applies the Grubbs' test for the outlier detection method.

The research is laid down for several drugs, which have a different historical pattern: one with shortage and another one without it. The author uses 13 weeks' time frame to predict the forecast and improve its accuracy. The author detects that products with uncertain sales have outliers.

The author tests four various approaches: the first approach uses aggregated sales of pharmacy chain for forecasting; the second approach uses aggregated sales for a pharmacy chain and response to outliers; the third approach uses sales by pharmacies for forecasting; the fourth method–sales by pharmacies and response to outliers.

The author tested four approaches for the pharmacy chain, which consists of 8 pharmacies and eight methods, which have seasonal and not seasonal patterns. The author delivers 280 tests to prove the forecasting method and its accuracy.

The other sections of this document are set out as follows: Sections Literature Review, Forecasting with Exponential Smoothing Methods, and Planning Levels provide a literature overview of different demand patterns, time series characteristics (6), and forecasting methods. Section Forecasting model presents the forecasting model, its structure, techniques for missing sales data and outlier detection, and the revision of forecasting accuracy. The application of the model is provided in Section Methodology. Later on, the tests and practical results are described in Section Test and Results. And finally, the relevant concluding remarks are presented in Section Discussion.

## Literature Review

There are many forecasting methods provided by the authors. Among those methods, priority is given to the adoption of forecasting methods, such as the Holt-Winters Multiplicative method [(7) p. 530], in medical care cases, especially in forecasting future events with seasonal and other causal changes. In Holt–Winters Multiplicative method, the components of the initial value are defined.

Thus, to combat a dynamic but uncertain environment, pharmacies have no choice but to accelerate their actions to meet the needs of patients in a timely and effective manner [(8), p. 375]. In this respect, the revision of forecasting methods must be taken into account.

Recently, many forecasting methods have been proposed, where the biggest group of methods belongs to quantitative methods. By using a qualitative approach, sophisticated mathematical methods are used for making the decision [(9) p. 404]. Among the quantitative techniques, scientists have shown an interest in exponential leveling methods which are simple, robust, and easy to use [(10) p. 619]. As a result, the researchers have developed several exponential equalization methods aiming to improve the accuracy of forecasts. All methods with the exponential procedure, follow the main principle which is applicable in constructing a forecast–it assesses future events by extrapolation of historical values [(11) p. 447]. However, in the case of similarities in principles, these exponential equalization methods also show specific distinguishing characteristics in their calculation processes, parameter calculations, and response to different time series components [(12) p. 245, (13) p. 67, (14, 15) p. 451]. For example, the single exponential leveling technique is perfect for time series that exhibit a changing average that does not have a seasonal component (16). Because, with a trend component, varying levels, and growth rates, Holt's trend correction method shows its superiority. Meanwhile, the overcomes the two above-stated Holt-Winters methods in time series, having a changing seasonality, level, and trend [(15) p. 451, (17) p. 395, (18) p. 380, (19) p. 34].

Nevertheless, the Holt–Winters methods incorporating such parameters, i.e., at a constant rate or increasing amount, are further classified as multiplicative and additive [(12) p. 245, (15) p. 451, (17) p. 395, (18) p. 380, (19) p. 34, (20) p. 284]. In the 1960s Winter [(21) p. 330] proposed a multiplicative Holt-Winter method, which was foreseen for seasonal variations, which swing around average values [(22) p. 32]. In addition, the average values will grow, as many Western European countries will undergo a major demographic transformation, driven by the retirement of the baby boomers and the possibility of a sudden increase in life expectancy and the drug consumption will grow in long-term perspective (23). Contrary to the method described before additive Holt-Winters method is chosen for time series where seasonal fluctuations do not swing at the same time as the mean value [(20) p. 284].

There are discussions among the authors about the seasonality aspect from a shorter-term perspective (calendar season or year). Rossignol et al. (24) suggested the method to identify seasonality. They propose to test if the seasonality exists by using the Google trend tool. They analyzed multiple time series of pharmaceuticals sales and Google search data seeking (1) to test if the seasonality in drugs sales exists or not, and (2) to determine its magnitude if seasonality exists. During the research, the author by comparing winter and summer search data identified an average increase of 8.7 per cent, which according to Mann-Whitney's test, shows that the *p*-value was lower than 0.01. Ayati et al. (3) reported that the increase in global demand for pharmaceuticals due to COVID-19 was evaluated as plus 8.9% in the March of 2020 with no seasonal effect consideration. Buxey (25) tells that sales peak is in November and December when public induced demand.

However, for incorporating level, trend, and seasonality in delivering forecasting, both forms of the Holt-Winters method have several equations, the suitability of which follows these components [(18) p. 380; (7) p. 360]. In this respect, the initial level and trend values are calculated using the smallest squares regression technique, the reading of still average changes adversely affects the accuracy of forecasts [(26) p. 56].

Upfront the forecast, the set of historical values is used for the calculation of components in a step-by-step way [(27) p. 3261]. In general, it is used in science, engineering, telecommunications, meteorology, finance, and economics [(28) p. 3860]. The exponential equalization methods are mostly used due to their strength [(12) p. 245; (29) p. 638], simplicity [(12) p. 245], and the low number of variables required [(8) p. 375, (12) p. 245]. Justifying the simplicity and easy application of this group of forecasting techniques [(8) p. 375; (11) p. 652; (4) p. 720] has attracted researchers to work on improvements [(30) p.1080].

Among these exponential equalization methods, the three most commonly accepted and literary techniques can be referred to as Holt's linear method [(14) p. 9], single exponential leveling [(13) p. 67], double or triple exponential leveling [(21) p. 330]. A single exponential one is known as a simple or easy application, and Holt's linear method provides forecasts for data with the trend. Although Holt's linear method has the highest quality, it misses its accuracy for data with seasonal patterns [(31) p. 750]. In such a case, the Holt–Winters additive and multiplicative methods bring forecasts which incorporate seasonality aspects [(32) p. 180].

There are two forms of Holt–Winters technique (i.e., addition and multiplication), the first one fits in a situation where is a stable pattern throughout the seasonal demand time frame, and another one with a dynamic adjustment feature is perfect for dynamic changes during the period. However, the application of these methods poses the challenges in setting the components for time series: level, trend, and seasonality; also finding the values for equalization constants, as well as using complex recursive calculation processes [(30) p. 1080, (32) p. 180, (33) p. 80]. Therefore, like other methods used for forecasting time series, several approaches were provided seeking higher accuracy of the usually applied Holt-Winters additive and multiplicative methods, and appropriate application cases.

For example, Grubb and Mason [(34) p. 80] adopted the Holt-Winter method to reach the forecast of United Kingdom air passengers and the results improved the level of accuracy (exactly, MSE). Lepojevic' and Andelkovic'-Pešic' [(35) p. 230] performed research where MSE is prioritized. On the other hand, Chatfield and Yar [(22) p. 32] applied components of level, trend, and seasonality, expecting to improve the accuracy of forecasts. Bermúdez et al. [(30, 32) p. 1080] also calculated the initial values for these three-time series parameters by reducing the forecast error.

Furthermore, Arora and Taylor [(36) p. 3240] apply the average historical data to be the starting values for the main components of the time series. Sudheer and Suseelatha [(37) p. 342] suggest using the average historical data for identifying the level, and the average historical data for the early two days for identifying the trend. Primary seasonal components shall be calculated by revising annual historical information. Costantino et al. [(38) p. 315] take average historical data to estimate the initial values of time series components.

The prediction of sales that is essential for drug supply (39), the Holt-Winters multiplicative method must be subject to research, when during the forecasting procedure its level, growth rate, and seasonal index are defined. The accuracy of the forecast depends on historical data [(7) p. 360; (26, 40)] and the results of other methods.

The author revised the papers applying forecasting methods for drug sales forecasting and provided the summary of results under Table 1. The papers were identified by using Google Scholars and OAister tools, the retrieved papers were printed in journals published by Elsevier, Taylor & Francis, IOP Science, IEEE, and IOP conferences, and hospital reports.

**Table 1:** The summary of methods applied for drugs sales forecasting.

**Model type**	**Method**	**Authors**
Methods with no seasonality	Single exponential smoothing	(41, 42)
	Moving average	(40, 41)
	Naive	(40)
	Holt's linear	This paper
	Holt-Winters no trend	This paper
	Non-seasonal ARIMA	(40, 43)
Methods with seasonality	Holt-Winters additive	(44)
	Holt-Winters multiplicative	(45)
	Double exponential smoothing	(46)
	Seasonal ARIMA (SARIMA)	(42)
Modified methods	Holt's linear with damping parameter	(47)
	Theta (Holt's linear with smoothing parameter)	(43)
	ETS (Double exponential smoothing with unpredictable change)	(43)
Hybrid methods	ARHOW (ARIMA & Holt-Winters additive)	(48)

The author identified that both Holt's linear and Holt-Winters no trend methods were not applied for forecasting drugs sales.

Restyana et al. (41) applied Single exponential smoothing and moving average for prescriptive drugs: paracetamol and vitamins, however, do not provide MAPE value.

Nikolopoulos et al. (40) prove the application of the naïve method for long-period forecasts which are built for generic pharmaceuticals and suggest applying non-seasonal ARIMA for branded drugs seeking to build one-year forecasts.

The seasonal part of the ARIMA model consists of terms that are similar to the non-seasonal components of the model, but involve backshifts of the seasonal period. However, ARIMA has a lot of model modifications: White noise ARIMA, Random walk ARIMA with no constant, Random walk ARIMA with a constant, Autoregression ARIMA, and Moving average ARIMA.

Ribeiro et al. (47) stay that Holt's linear forecasting function tends to overestimate that's why it is suggested to use its modification and use SMAPE to present the error size, which vary for alfa = 0.2 from to 1.27 to 1.29.

Newberne (44) analyses Holt–Winters additive model for pharmaceuticals and reaches MAPE which is equal for alfa = 0.2 to 14.59%.

Bon and Ng (45) used Holt-Winters multiplicative to forecast inventory demand at the healthcare center. And reached the lowest RMSE.

Zahra et al. (42) compared SARIMA with single exponential smoothing and presented that MAPE values are 13 and14 percent accordingly.

Exponential smoothing and ARIMA models are the two most widely used approaches to time series forecasting (49). Further on, the author revises forecasting with exponential smoothing.

## Forecasting With Exponential Smoothing Methods

Being reliable, the exponential smoothing methods are quite simple forecasting techniques that are used to smooth time series without the need of becoming a parametric model. It is based on a recursive calculation scheme, in which forecasts will be revised for each new observation. Although exponential smoothing sometimes serves as a simple prediction method (i.e., naive), it performs well in practice. Therefore, these methods are often used in business practice to forecast inventory requirements. Besides, compared to more sophisticated techniques forecasting techniques, these exponential smoothing methods have also performed remarkably well in the forecasting process (50, 51).

In general, single exponential, trend-corrected exponential, and Holt–Winters methods with seasonal are considered three basic types of exponential smoothing (13, 14, 21). The main feature of this type of forecasting method is as such: (i) time series are revised to create the equation of unobserved three components (i.e., level, trend, and seasonality); and (ii) these components need to be changed over time if the demand pattern has some structural changes. According to Hyndman et al. [(15) p. 440], the addition or multiplication operators can serve for a combination of these components up to 24 variations of exponential smoothing methods.

Producing short-range forecasts is the best way of simple smoothing application, usually only one or two forthcoming months. The assumption that the data fluctuates around a relatively stable mean (no trend or consistent growth pattern) is the critical point in the model. A weighted average of the present time series and the previous smoothed ones serve as a new smoothed forecasted evaluation; the last smoothed observation is derived from the previously observed value and the smoothed evaluation before the previous one, and so on.

A trend usually is a subject of double exponential smoothing which is quite similar to simple smoothing, thought two components–level and direction–thereof need to be updated each period. In the initial stage, it is a smooth estimate of the value of the data that should be performed at the end of each period. But in the case of trend estimation, it goes like a smooth estimation of average growth.

The method that deals with trend and seasonality at the same time is named Triple Exponential Smoothing. To cope with seasonality, it includes one more parameter–a third component that deals with seasonality.

To construct the forecast, most of the techniques require the stationarity requirements to be satisfied. Time series are the first-order stationary if the expected value of *Y(t)* remains the same for all *t*. For example, a process is first-order stationery when we eliminate any sort of the trend by some operations such as differencing. A time series is a second-order stationery if it is first-order stationary and covariance between *Y(t)* and *Y(i)* is a function of length (*i-t*) only. Also, a process is a second-order stationary when we also stabilize its variance by some transformation, for example, such as taking a logarithmic scale.

The exponential smoothing approach is often used for data forecasting that includes seasonality, changing trends, and seasonal correlation. Also, being developed as an extension of exponential smoothing for directions and annual time series, this method might be referred to as double exponential smoothing.

There are various forecasting methods. Two of them relate to the concept of pharmacies: space-driven and demand-driven. Demand-driven forecast involves the prognosis of the number of patients, penetration of drugs and average quantity.

The equations for demand forecasts are usually such as


$$Y_{t+1}\;=\;P_{t+1}\;\times \;D\;\times \;\underline{Y_{t}}$$

where *P* is the forecast of the number of patients visiting the pharmacy (% of number of patients treated as a part of a number of patients prescribed), coefficient *D*—penetration of drug, Y – average sales quantity.

If we know the number of patients and the penetration of drugs, such a method could be applied. In the case of prescribed medications, there is essential to understand how many patients are defined and how many of them have medical treatment.

In this particular case, the number of patients, which are registered with Hemophilia A, Hemophilia B, von Willebrand disease, and Factor VII deficiency is growing. Still, the number of patients treated for the above-mentioned disease is almost stable.

However, in other categories, the sales of drugs are increasing or decreasing from a year-to-year perspective. By using statistics reported by the State Medicines Control Agency of Lithuania (52) for the Baltic countries, it is evident that sales in some categories are increasing:

– Sales for drugs prescribed for alimentary tract and metabolism (1st level, anatomical main group): drugs for acid-related disorders, drugs for peptic ulcer and gastro-esophageal reflux disease, antiemetics, and antinauseants are increasing by 6% 2018 year. By using historical consumption to forecast sales it is recommended to add safety stock to cover the increase rate, which is 6%.
– Sales for drugs prescribed for the respiratory system: nasal preparations and throat preparations are increasing by 4% in 201. By using historical consumption to forecast sales it is recommended to add safety stock to cover the increase rate, i.e., 4 percent.
– Sales for drugs prescribed for the cardiovascular system are increasing by 1% in 2018 year. By using historical consumption to forecast sales it is recommended to add safety stock to cover the increase rate, i.e., 1%.
– Sales for prescribed dermatological: antifungals for dermatological use and anti-acne preparations are increasing by 10% 2018 year. By using historical consumption to forecast sales it is recommended to add safety stock to cover the increase rate, i.e., 10 percent.

However, if there is no information mentioned above, it is better to apply forecast methods incorporating sales data of previous historical periods.

The demand aggregation level is also especially important. Herein, the demand pattern of drugs fits unimodal statistical distribution, and the Jarque–Bera normality test shows (exactly probability which is 0.1) that the hypothesis that data of drugs are distributed according to the normal distribution, cannot be rejected. Even though, it is still a question at which level in pharmacy chain generated forecast is the most accurate one.

## Planning Levels

It is very important to anticipate the right demand at every pharmacy as it helps to plan inventory and better distribution of products in all selling points.

In order to show some of the key differences in pharmacy chains the aggregated demand (for pharma chain) and disaggregated demand (for individual pharmacies) is considered. In the aggregation case, the sales of multiple selling locations are summed and demand is forecasted on the chain level.

According to Kanyalkar and Adil (53), the number of studies on aggregation/disaggregation in the context of planning is limited. Kanyalkar and Adil (53) suggest applying hierarchical planning approach covering the integration of planning decisions. The authors highlighted that the type of aggregation used in a chain case and parallel multiple outlets case may be different. When it is planned in a disaggregated way further the integration of plans generated at multiple selling locations level is necessary, in terms of time horizon (54), time periods, and volumes per product. This integration could be weighted and these weights could be specified by experts but the weight represent average value across all products, not the values that focus on separate product specifics and sales distribution across different pharmacies. In general, the naive forecast also often outperforms more complicated point aggregation schemes, such as weighted combinations (55). The average could be selected between the lowest and the highest sales numbers which are represented by different pharmacies. The forecast generated by taking their average performs better than choosing one of the two experts at random; when the estimates of experts perform equally as well as the average expert. Only when the crowd of forecasts being combined has a high degree of dispersion in expertise, some individuals in the crowd might stand out, and in such cases, there could be some benefits to chasing a single expert forecaster instead of relying on the entire crowd. When it is better to select the minimum level of experts or search for other solutions (Table 2) (55).

**Table 2:** The summary of studies on hierarchical planning approach.

**Studies**	**Planning levels**	**Dimensions used for planning**
Kanyalkar and Adil (53)	Two	Time
Katayama (56)	Two	Product, time
Mehra et al. (57)	Two	Product, time
Moreira and Oliveira (58)	Two	Product, time
Leong et al. (59)	Three	Product, time
Tsubone and Sugawara (60)	Three	Product

The author provides the summary of studies dedicated to the hierarchical planning approach, where the most dominating dimensions used by the authors are product and time. Most of these studies apply hierarchical planning integration in other industries than the pharmaceutical one, herein dominates production industry cases. Kanyalkar and Adil (53) specify that aggregated plan could be prepared on the country level and later on, could be separated between individual selling locations. Or vice versa first could be generated disaggregated forecasting plans and later they could be integrated.

Two planning levels mean that the first level is dedicated to a single pharmacy and the second level shows the integration of plans on the pharmacy chain level. For disaggregated and aggregated level planning Kanyalkar and Adil (53) suggest using weekly demand forecasting both for selling locations and for the country level cases. Based on these investigations, the author builds a forecasting model.

## Forecasting Model

The proposed forecasting model consists of several successive steps such as setup of historical data, selection of product for testing when the application of approaches, detection of outliers and missing sales data, the application of methods, and the estimation of accuracy of forecasts. All the above-mentioned steps are presented in the following subsections.

Below, in Section The Structure of the Model, the author presents possible approaches, further on, in the chapter Techniques for Handling Missing Data author gives information on missing data setup, and in Outliers Detection–provides information on outlier detection, Section Forecasting Methods describes forecasting methods, and finally, in Section Forecasting Accuracy there is the presentation of the evaluation of forecasting accuracy.

### The Structure of the Model

The forecasting model consists of four approaches, which in the model are applied sequentially.

1. The first approach uses aggregated sales of pharmacy chain for forecasting:

$$Y_{t}=\sum_{i=1}^{n}Y_{i}(t)$$

2. The second approach uses aggregated sales for the pharmacy chain and responds to outliers:

$$Y_{t}=\sum_{i=1}^{n}Y_{i}(t),\;Y_{t}\epsilon (Y_{t},3\times s)$$

3. The third approach uses sales by pharmacies for forecasting and aggregated forecasts for the pharmacy chain:

$$\begin{array}{l} Y_{i}=\left\{Y_{ij},j\epsilon t,i\epsilon n\right\} \end{array}$$

Forecasts are calculated according to formulas placed under **Figure 2**, and later these forecasts are summed:

$$Y_{t+\tau }=\sum_{i=1}^{n}Y_{i}(t+\tau )$$

4. Fourth approach–sales by pharmacies, responses to outliers and aggregated forecasts for pharmacy chain:

$$Y_{i}=\{Y_{ij},Y\epsilon (\underline{Y_{t}},3\times s),j\epsilon t,i\epsilon n\}$$

Forecasts are calculated according to formulas placed under **Figure 2**, and later these forecasts are summed to get *Y*_*t*+τ_.

Where *n* is the number of pharmacies and is equal to 8, *t*—week number and is the same as 13, τ**—**index of the upcoming week, *s*—standard deviation.

Before the application of the third and fourth approaches for a product with shortage, missing data (i.e., data sets with zero values) are replaced with Mean values. The placement of missing values is mandatory action before applying outlier detection tests.

### Techniques for Handling Missing Data

Missing data reduces the representativeness of the sample and may skew conclusions about the demand. In general, there are three main ways to handle missing data: (1) allegation—when values are filled in place of missing data, (2) omission —when samples with invalid data are excluded from further analysis, and (3) report—by direct application of methods not affected by missing data values.

With some practical applications, experimenters can control the level of missing data and prevent missing values before making a decision. For example, it is often not possible to step to outlier detection until the missing data is not handled. The allegation must be selected. Otherwise, the next step cannot be processed; thus, a period with missing data can be removed because one product in the pharmacy chain was in shortage and was not timely supplied to patients. In research studies, it is common to reduce the negative impact of missing data.

In cases where values are likely to be missing, the researcher is often advised to plan to use data analysis techniques that will cause little or no biased conclusion about the potential demand. Further on, the missing data is inputted for shortage and recovery period upfront the outlier detection procedure setup.

### Outliers Detection

The presence of outliers in the sales data can significantly bias a forecast. Outlier detection is especially critical for making predictions based on historical data where the size of sales is important and could lead to overestimation or underestimation. This especially is the case in panic buying of popular product which was under the shortage for some period. The literature review points to several studies using outlier detection methods for drug sales data cleaning. The demand change, which leads to a shortage, in the case of induced demand has to be reviewed and evaluated from the significance of sales point of view. The cleaning of sales data for each product by using the outlier detection method has to be performed before the application of the forecasting methods. Ribeiroa et al. (47) use the Dixon outlier detection method seeking to avoid abnormal sales and out-of-stocks in pharmacies.

There are various outlier test methods, like Grubbs' test (1950), Dixon test (1953), David test (1961), Barnett and Lewis test (1994), and Rosner's analysis (2011). Some of these tests face the problem of misleading situations.

The Grubbs' test is used to detect deviations above the normal distribution limits [(61) p. 5]. The result is a probability indicating the basic data of actual sales. Using the outliers test method, the difference between the sample mean and the extreme data concerning the standard deviation can be plotted. The test determines one external value simultaneously with different probabilities than a data set with an assumed normal distribution.

Luo and Cisler (54) tried the Grubbs test for detecting outliers for drugs helping to prevent cancer disease and had positive outcomes [(54) p. 213].

Based on approximate data, the author uses this test to reduce the number of extraordinary sales and to evaluate outliers according to the Grubbs' test method.

The application of the Grubbs' test has practical value and could be used for the definition of potential demand data sets to avoid under-supply cases.

The detection of outliers using the Grubbs test and the evaluation of data points helps to streamline historical sales data. In case an outlier is detected for sales points outside healthy distribution boundaries, sales data inside healthy distribution boundaries are taken.

There is a test for the null hypothesis that the data has no outliers vs. the alternative hypothesis that the minimum or maximum value is an outlier. There are three steps to proceed with outlier detection:

Step 1. Sorting of data points from smallest to largest;
Step 2. Finding the mean (*Y*_*t*_) and standard deviation (*s*) of the data set;
Step 3. Calculating the *G* test statistic for a two-tailed test using one of the following equations:

$$G=\frac{\max\;i=1,\ldots ,N\;|Y_{i}-\underline{Y_{t}}|}{s}$$

The *G* test statistic for maximum value in the data set may be an outlier; it is better to use the test statistic (right-tailed test): $G=\frac{\left|Y_{\max}-\underline{Y_{t}}\right|}{s}$.

In case, the minimum value in the data set may be an outlier; it is better to use the test statistic (left-tailed test):


$$G=\frac{\left|\underline{Y_{t}}-Y_{\min}\right|}{s}$$

The critical value for the test is


$$\begin{array}{l} G_{crit}=\frac{N-1}{\sqrt{N}}\sqrt{\frac{t_{\alpha /\left(2N\right),N-2}^{2}}{N-2+t_{\alpha /\left(2N\right),N-2}^{2}}} \end{array}$$

Where *t*_α/(2*N*), *N*−2_ is the upper critical value of a t-distribution with N-2 degrees of freedom, when the alpha (α) representing significance level is equal to 0.05.

If calculated *G* is more significant than *G*_*crit*_, the data point is not an outlier, and the null hypothesis H0 is accepted. Overwise, if *G* is lower than *G*_*crit*_, the second hypothesis is accepted.

**Figure 1:**
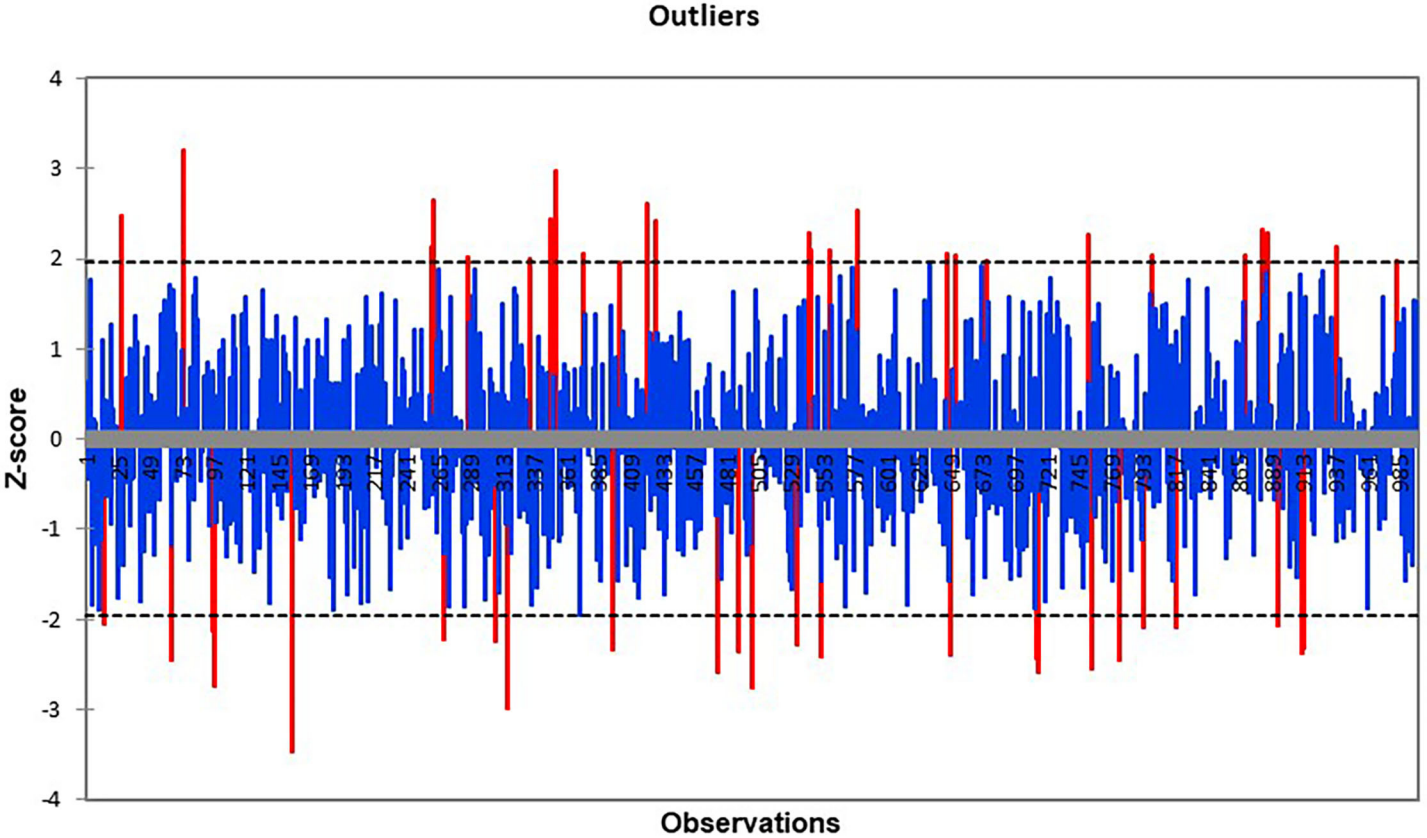
Grubbs test.

After the procedure of outlier detection (Figure 1), it is necessary to set up a *3s* value instead of an outlier value. The process helps to avoid upstream and downstream demand, which is represented by historical data points or oversupply and undersupply cases, which are present in case of wrong forecasts.

### Forecasting Methods

In this paper author applies forecasting methods and research their accuracy, to define which of those methods per each approach must be recommended for practical applications.

There is a variety of more sophisticated methods concerning seasonality: no, additive, or multiplicative.

Eight methods are separated into two categories: (1) methods with no seasonality and (2) methods with seasonality.

From the methods mentioned in Table 1, the author uses five methods with no seasonality: single exponential smoothing, moving average, naive, Holt's linear, and Holt-Winters no trend. These are shortly presented below:

1. Single exponential smoothing method applies weighted averages, where the lowest weights are associated with the oldest observations, and these weights decrease exponentially. The setup of weights also lowers the increase in variability [(62) p. 269].
2. Moving average method is applicable for trend-based estimation. It is used to estimate the expected value of demand and the standard deviation [(39) p. 8934].
3. The naive method assumes that all predictions are the value of the last observation. The prediction of a random walk, called naive here, is a primary benchmark that does not require parameter identification and should be surpassed by more sophisticated methods for added complexity [(63) p. 28].
4. Holt‘s Linear method is developed based on single exponential smoothing and is taken to predict trend data. This method includes a predicted equation and two smoothing equations (one for the level, the other for the trend). The very small value of the trend component means that it is near to the flat. So, usually, this component must be higher.
5. Holt-Winters no trend method has seasonal variation but has no trend. However, it uses more than just one parameter, although the trend component in this method is not taken into account.

From the methods mentioned in Table 1, the author uses three methods with seasonality components: (1) Holt-Winters additive; (2) Holt-Winters multiplicative; and (3) double exponential smoothing. These are shortly presented below:

1. Holt-Winters Additive method has additive seasonal variation and a linear time trend. This method handles more effectively additive seasonality [(64) p. 162].
2. Holt-Winters Multiplicative Method Has Multiplicative Seasonal Variation and Linear Time Trend. It Is the Assumption That the Time Series Is Formed From Unattended Components: Level, Direction, and Seasonality (65). These Components Need to be Adapted Over Time, as They Must Follow the Demand Pattern [(66) p. 162].
3. Double Exponential Smoothing Method Is Developed for Time Series With Multiplicative Seasonal Variation. Herein, the Components Were Smoothed Using Different Parameters Separately, Namely *a* and *c*.

**Figure 2:**
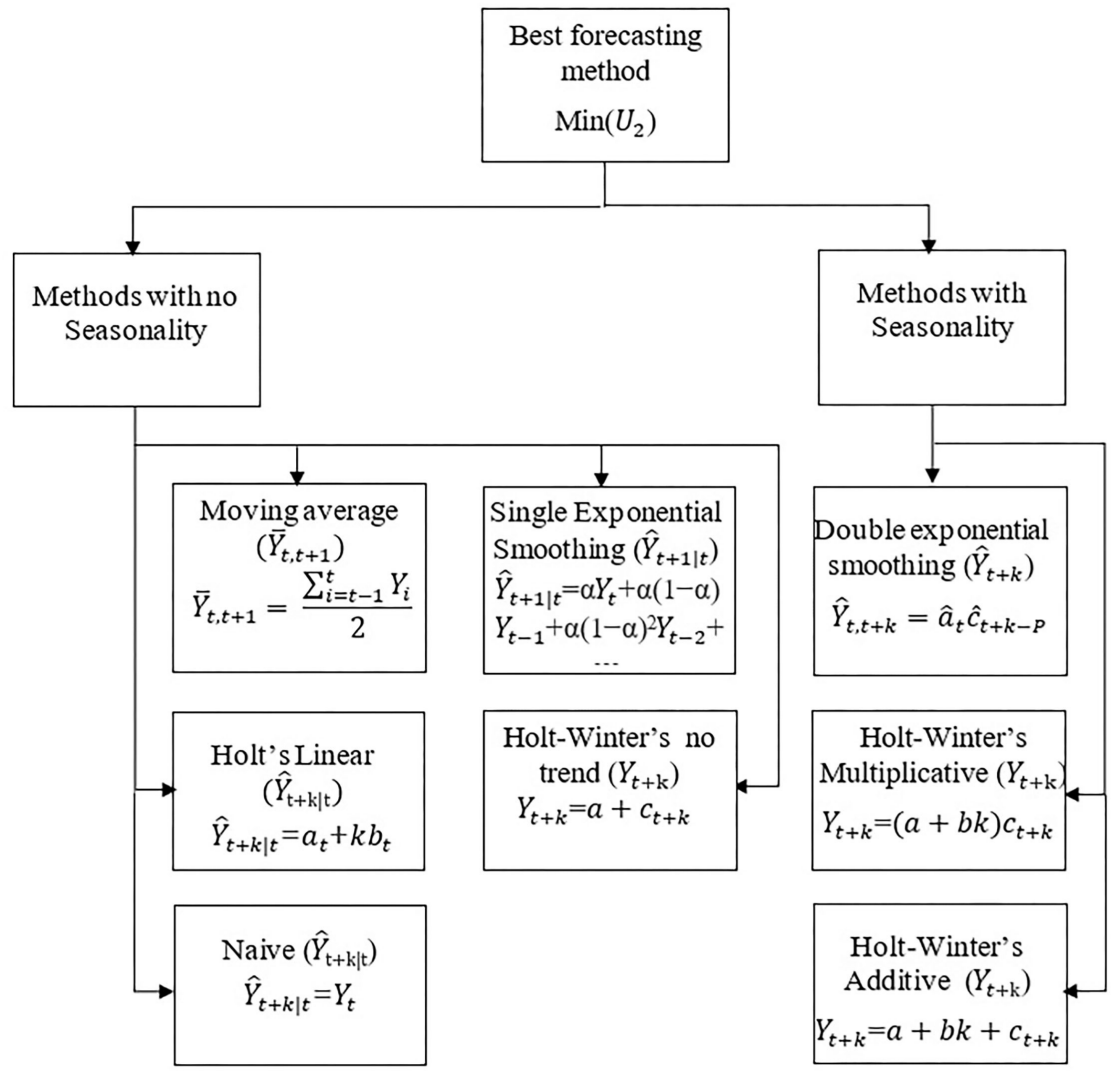
Selection of appropriate forecasting methods.

Below author defines the mathematics of forecasting methods and displays it in Figure 2.

Where *a*_*t*_ denotes the value of the level component (intercept) at time *t*, *b*_*t*_ - an estimate of the trend (slope) at time *t*, α is the smoothing parameter and represents values between 0 ≤ α ≤ 1, *c*_*t*_-a multiplicative or additive seasonal factor; *k*>0 and is equal to 1,2…; and *P* is the number of periods within the seasonality.

Finally, the decision must be taken regarding the method, which suits the best and has the lowest forecasting accuracy.

### Forecasting Accuracy

There are many measures for forecasting accuracy: Bias, SE, MSE, MAE, RMSE, and MAPE. There mean squared error (MSE) is a combination of bias (Bias) and standard forecast error (SE). MAE, RMSE and MAPE compare actual values and forecasted values, which are predicted with the model application [(66) p. 5]. Inaccuracy calculated after estimating the root mean square error (RMSE) or the mean absolute prediction error (MAPE) was generally reported as worse in Brooks et al. study [(67) p. 1295]. Because RMSE and MAPE are used to measure the deviation from the expected and actual values, the values closer to 0 are better [(68) p. 8].

Qian et al. (69) developed forecasting models, for which evaluation some measures were used MAE, RMSE, and MAPE. Authors mention, that in case the results of prediction are inconsistent, MAPE is often selected for a benchmark due to its stability [(69) p. 4].


$$\begin{array}{l} MAPE\;=\;\frac{1}{t}\sum_{i=1}^{t}\frac{\left|Y-Y_{i}\right|}{Y} \end{array}$$

Where *Y* and *Y*_*i*_ are the actual and predicted values, respectively; *t* is the number of data samples linked to time-period.

Theil's U_2_ measures forecast quality by comparing the forecast results of the applied method with the naive forecast.

The best methods are selected based on forecasting accuracy Theil's U_2_ statistics:


$$U_{2}=\frac{MSE(applicable\;method)}{MSE(naive\;method)}$$

where *MSE* is forecasting evaluation statistics, which is defined such as:


$$MSE=\frac{1}{t}\sum_{i=1}^{t}{\left({\hat{Y}}_{i}-Y_{i}\right)}^{2}$$

For the identification of the proper forecast method, U_2_ value must be as lower as possible.

In such case U_2_ = 1 for the naive method; U_2_ <1 when the results of the applied forecasting method are better than the effects of the naive method; U_2_ > 1 when the results of the used forecasting method are worse than the results of the naive method, i.e., naive method brings better results.

It is no case when *U*_2_ statistics is applied in forecasting the demand for drugs or the number of medical services but is quite often used in macroeconomic forecasting.

## Methodology

The author applies a forecasting model that integrated various methods into the statistical framework. By using the model, the author delivers 280 tests: 144 tests for the product without shortage and 136 tests for the product with the shortage. Among the methods, all of them have the same number of tests–the application of each method reaches 35 tests. Traditionally, the exponential smoothing methods go without reference to a statistical framework as other methods can be employed to resolve the problem of method selection. Herein these methods are applied in combination with other methods and are linked to a statistical framework.

For the validation of prediction [(70) p. 37], the sample shall be divided into two parts: the installation sample and the inspection sample. A suitable example is used to find reasonable values for the components, often by specifying the sum of the squares that provides the forecast error criterion one step ahead. The installation sample is used to evaluate the prediction capabilities of the method by a method such as MAPE. And the inspection sample uses a standard version of exponential smoothing based on the hypothesis that it is effectively reduced to the appropriate embedded solution when data require it. Herein the empirical part is dedicated to the inspection sample and hypotheses are formulated to select the most accurate forecasting ways and methods, when data of selected prescriptive products are distributed according to normal distribution.

Despite that many methods are applied to solve the problem, and the application of Holt-Winters method is usually selected for yearly time series, herein it is applied for weekly data forecasting.

Another aim is to evaluate the effectiveness of these traditional methods in terms of the information criteria method chosen for the process. For the evaluation of forecasting, accuracy author uses Theil's U_2_, which outperforms MAPE.

All these tests and results, including forecasting accuracy, are presented in the section below.

## Test and Results

**Figure 3:**
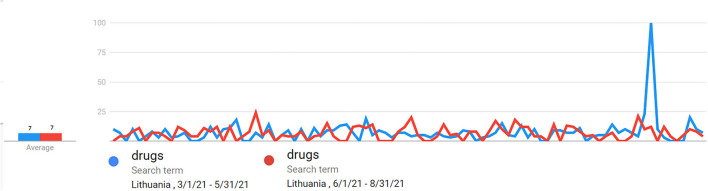
Seasonality impact check.

In general, the seasonality of products aspect could be checked by using Google trends tool representing drugs search results on daily basis. The author has checked the spring and summer search results in Lithuania during 2021 and found that during the pandemic the behavior of consumers eliminated the seasonality aspect (Figure 3).

But after the pandemic, knowing the impact of seasonality on sales the volume adjustment actions should be taken.

### The Application of Forecasting Methods

All components representing level, trend, and seasonality must be between 0 and 1. The smaller the parameters, the more modest increase in variability.

For the practical application of methods, the values are defined in Table 3.

**Table 3:** The values in components.

**Methods**	**Values**	**Methods**	**Values**
Single exponential smoothing method	a = 0.2	Holt-Winter‘s no trend method	a = 0.2, c = 0.05
Moving average method	interval is equal to 2	Holt-Winter‘s additive method	a = 0.2, b = 0.15, c = 0.05
Naive method	interval is equal to 1	Holt-Winter‘s multiplicative method	a = 0.2, b = 0.15, c = 0.05
Holt‘s linear method	a = 0.4, b = 0.7	Double exponential smoothing method	a = 0.2, c = 0.15

The above-stated values are constant for all tests that are delivered.

### The Results of Approaches

First, the author starts testing approaches and forecasting methods for the product without shortage.

The results for the first approach and product without shortage are presented in Table 4.

**Table 4:** The results of forecasting (first approach and product without shortage).

***T***	***Y***_***t***_	**Single expo. smooth. (${\hat{Y}}_{t+1|t}$)**	**Double expo. smooth. (${\hat{Y}}_{t+k}$)**	**Moving average (${\bar{Y}}_{t,\;t+1}$)**	**Holt's Linear (${\hat{Y}}_{t+k|t}$)**	**Holt-Winter's Multip. (*Y*_*t*+*k*_)**	**Holt-Winter's Additive (*Y*_*t*+*k*_)**	**Holt-Winter's no trend (*Y*_*t*+*k*_)**	**Naive (${\hat{Y}}_{t+k|t}$)**
1	21819	N/A	22496	N/A	22496	21739	21739	21883	N/A
2	22344	21819	22496	N/A	22496	21856	21856	21867	21819
3	20675	21924	21794	22081	21725	22086	22086	21986	22344
4	22695	21674	20865	21509	20301	21818	21818	21658	20675
5	18226	21878	20581	21685	20925	22125	22125	21917	22695
6	19217	21147	19389	20460	18755	21169	21169	20994	18226
7	17944	20761	18628	18721	17979	20622	20622	20550	19217
8	17799	20198	17745	18580	16995	19822	19820	19898	17944
9	18986	19718	17010	17871	16571	19119	19115	19373	17799
10	24854	19571	16720	18392	17467	18865	18860	19276	18986
11	16402	20628	17905	21920	22421	20260	20255	20671	24854
12	18272	19783	17117	20628	20327	19177	19171	19603	16402
13	19393	19480	16896	17337	19243	18776	18769	19270	18272
Forecast accuracy									
MAPE		10.98	8.21	12.29	10.19	9.81	9.81	9.81	12.86
MSE		6852580	7001301	9211742	8947946	6558724	6558831	6329627	11814118
U_2_		0.58003	0.59262	0.77972	0.75739	0.55516	0.55517	0.53577	1.0

All forecasted values presented in Table 4 are not rounded integer values; even though there are some numbers after the comma.

From forecasting statistics, it is evident that the best forecasting results are delivered by Holt-Winters Multiplicative method.

The results for the second approach and product without shortage are presented in Table 5.

**Table 5:** The results of forecasting (second approach and product without shortage).

**Forecasting accuracy**	**Single expo. smooth**.	**Double expo. smooth**.	**Moving average**	**Holt's Linear**	**Holt-Winter's Multip**.	**Holt-Winter's Additive**	**Holt-Winter's no trend**	**Naive**
MAPE	10.49	8.03	11.44	9.50	9.39	9.39	9.40	12.04
MSE	5827558	5830398	7362487	7070061	5498635	5498758	5305605	9473552
U_2_	0.61514	0.61544	0.77716	0.74629	0.58042	0.58043	0.56004	1.0

From this test, it is evident that *U*_2_ is better for moving average and Holt's Linear methods when in the first approach, but *U*_2_ results for other methods are worse.

The results for the third approach and product without shortage are presented in Table 6.

**Table 6:** The results of forecasting (third approach and product without shortage).

**Forecasting accuracy**	**Single expo. smooth**.	**Double expo. smooth**.	**Moving average**	**Holt's Linear**	**Holt-Winter's Multip**.	**Holt-Winter's Additive**	**Holt-Winter's no trend**	**Naive**
MAPE	10.98	8.21	12.29	10.19	9.77	9.81	9.81	12.86
MSE	6852580	7001301	9211742	8947946	6551488	6558831	6329627	11814118
U_2_	0.58003	0.59262	0.77972	0.75739	0.55455	0.55517	0.53577	1.0

The best forecasting results are delivered by Holt-Winters Multiplicative method, and they are better than in the first approach, and other methods have the same *U*_2_ values. The results show that forecasting by individual pharmacies helps to improve the forecast. Herein Theil's U_2_ test shows that even though according to the MAPE, the double exponential smoothing method outperforms Holt-Winters Multiplicative method, the highest accuracy is bringing Holt-Winters Multiplicative method. The results for the fourth approach and product without shortage are presented in Table 7.

**Table 7:** The results of forecasting (fourth approach and product without shortage).

**Forecasting accuracy**	**Single expo. smooth**.	**Double expo. smooth**.	**Moving average**	**Holt's Linear**	**Holt-Winter's multip**.	**Holt-Winter's additive**	**Holt-Winter's no trend**	**Naive**
MAPE	12.37	7.21	10.53	8.36	8.70	8.73	8.65	9.98
MSE	7173227	4218869	5729728	5451635	4393754	4404285	4224950	6787283
U_2_	1.05686	0.62158	0.84419	0.80321	0.64735	0.64890	0.62248	1.0

The latter approach brings the worst *U*_2_ results. So, the best way is to apply the third approach for the product without shortage.

Second, the author starts testing approaches and forecasting methods for the product with the shortage. The data sets with shortage are adjusted to the value of 0.0001 instead of null values.

The results for the first approach and a product with a shortage are presented in Table 8.

**Table 8:** The results of forecasting (first approach and product with shortage).

***t***	***Y***_***t***_	**Single expo. smooth. (${\hat{Y}}_{t+1|t}$)**	**Double expo. smooth. (${\hat{Y}}_{t+k}$)**	**Moving average (${\bar{Y}}_{t,\;t+1}$)**	**Holt's linear (${\hat{Y}}_{t+k|t}$)**	**Holt-Winter's multip. (*Y*_*t*+*k*_)**	**Holt-Winter's additive (*Y*_*t*+*k*_)**	**Holt-Winter's no trend (*Y*_*t*+*k*_)**	**Naive (${\hat{Y}}_{t+k|t}$)**
1	3037	N/A	3037	N/A	3037	3005	3005	2874	N/A
2	2910	3037	3037	N/A	3037	2926	2926	2914	3037
3	2751	3011	2882	2973	2825	2835	2835	2913	2910
4	2799	2959	2722	2830	2613	2726	2725	2873	2751
5	2375	2927	2606	2775	2557	2656	2656	2854	2799
6	2469	2816	2422	2587	2303	2493	2493	2734	2375
7	2530	2747	2295	2422	2234	2391	2391	2668	2469
8	0.0001	2703	2212	2499	2300	2332	2332	2633	2530
9	0.0001	2163	1573	1265	684	1619	1610	1975	0,0001
10	10	1730	1015	0	−477	1038	1013	1481	0,0001
11	1794	1386	541	5	−1033	579	532	1113	10
12	1449	1467	556	902	137	684	632	1283	1794
13	1846	1464	525	1621	1069	699	650	1325	1449
Forecast accuracy									
MAPE		1731	935	31	229595084	953	931	1349	21106
MSE		1320448	972673	1057640	1270897	969960	987844	1087908	841361
U_2_		1.56	1.15	1.25	1.51	1.15	1.17	1.29	1.0

The forecasting results show that the application of the naive method is the best as *U*_2_ is higher than 1 for other methods.

Before the application of the third and fourth approaches, the data sets for shortage weeks (i.e., the null values) in drug supply must be replaced with average values (*Y*_*t*_ *or* *Y*_*it*_) before the application of forecasting methods.

Summary statistics before treatment and after treatment are presented in Table 9.

**Table 9:** Summary statistics before treatment and after treatment (product with shortage).

**Summary statistics (Before treatment)**							
**Variable**	**Observations**	**Observations with missing data**	**Observations without missing data**	**Min**	**Max**	**Mean**	**Standard deviation**
Pharma1	13	3	10	127	383	296	82.99
Pharma2	13	3	10	212	406	318.4	64,48
Pharma3	13	3	10	143	432	306.1	93.01
Pharma4	13	3	10	194	375	320.1	54.66
Pharma5	13	3	10	167	384	286.1	77.71
Pharma6	13	3	10	149	376	286.4	81.51
Pharma7	13	3	10	217	397	309.4	59.42
Pharma8	13	2	11	10	383	248.7	128.12
**Summary statistics (After treatment)**							
**Variable**	**Observations**	**Observations with missing data**	**Observations without missing data**	**Min**	**Max**	**Mean**	**Standard deviation**
Pharma1	13	0	13	127	383	296	71.87
Pharma2	13	0	13	212	406	318.4	55.84
Pharma3	13	0	13	143	432	306.1	80.55
Pharma4	13	0	13	194	375	320.1	47.34
Pharma5	13	0	13	167	384	286.1	67.30
Pharma6	13	0	13	149	376	286.4	70.58
Pharma7	13	0	13	217	397	309.4	51.46
Pharma8	13	0	13	10	383	248.7	116.95

The results for the second approach and the product with a shortage are presented in Table 10.

**Table 10:** The results of forecasting (second approach and product with shortage).

**Forecasting accuracy**	**Single expo. smooth**.	**Double expo. smooth**.	**Moving average**	**Holt's Linear**	**Holt-Winter's Multip**.	**Holt-Winter's Additive**	**Holt-Winter's no trend**	**Naive**
MAPE	15.86	7.11	9.27	8.00	6.01	6.02	11.86	7.06
MSE	152158	35305	65323	49069	26876	26934	100024	52759
U_2_	2.88405	0.66918	1.23815	0.93006	0.50941	0.51051	1.89589	1.0

The best forecasting results are delivered by Holt-Winters Multiplicative method, and they are better than in the first approach. Such shows that forecasting by individual pharmacies helps to improve the forecast. However, the naive method is better than the application of single exponential smoothing, moving average and Holt-Winters no trend methods.

The third approach is not tested as from Table 8; it is evident that forecasts due to shortage which appeared in drug supply, have minus values for some weeks.

The results for the fourth approach and the product with a shortage are presented in Table 11.

**Table 11:** The results of forecasting (fourth approach and product with shortage).

**Forecasting accuracy**	**Single expo. smooth**.	**Double expo. smooth**.	**Moving average**	**Holt's Linear**	**Holt-Winter's Multip**.	**Holt-Winter's Additive**	**Holt-Winter's no trend**	**Naive**
MAPE	15.86	6.88	9.27	8.00	6.01	6.02	11.86	7.06
MSE	152158	30656	65323	49069	26763	26934	100024	52759
U_2_	2.88405	0.58107	1.23815	0.93006	0.50728	0.51051	1.89589	1.0

The latter approach brings the best *U*_2_ results for Holt-Winters Multiplicative method and one other method (i.e., Double exponential smoothing). So, the best way is to apply the fourth approach for the product with a shortage during supply. However, the naive method is better in the three previously mentioned cases.

The results per product are different, in case of shortage, the best way is to apply the fourth approach, and in case of the product without shortage—the third approach. Such must be used in delivering forecasts. Results prove that detection of outliers must be incorporated in the forecasting model to have procedures for products with a shortage in supply.

### Detection of Outliers

The chart below represents the detection of outliers, which are defined using the Grubbs' test method By using Grubbs' method outlier is detected in week 10 when the *G* value is 2.089 for the product without shortage in supply. There is also evidence that a product with a shortage has no outliers detected, but it has two shortage weeks and one recovery week. The results of outliers are summarized in Table 12.

**Table 12:** The results of Grubbs' tests by-products and accumulated sales (product with shortage).

**Outlier**	**G**	***G_***crit***_***	**Significance**
24854	2.089	2.412	yes

An alternative hypothesis for this case presented in Table 12 is accepted.

By using Grubbs' test, outliers are detected in pharmacies 2, 5, and 7 for the product without shortage in supply. The results of outliers are summarized in Table 13.

**Table 13:** The results of Grubbs' tests by pharmacies (product without shortage).

**Pharmacy**	**Outlier**	**G**	***G_***crit***_***	**Significance**
1	-	1.901	2.462	Yes
2	1,877	2.147	2.462	Yes
3	-	1.833	2.462	No
4	-	1.741	2.462	No
5	3,271	2.076	2.462	Yes
6	-	1.756	2.462	No
7	1,249	2.351	2.462	Yes
8	-	1.431	2.462	No

An alternative hypothesis for this case presented in Table 13 is accepted.

The results of Grubbs' test for the product with a shortage in supply are presented in Table 14.

**Table 14:** The results of Grubbs' tests by pharmacies (product with shortage).

**Pharmacy**	**Outlier**	**G**	***G_***crit***_***	**Significance**
1	127	2.364	2.462	Yes
2	-	1.905	2.462	No
3	143	2.007	2.462	Yes
4	194	2.664	2.462	Yes
5	-	1.770	2.462	No
6	-	1.946	2.462	No
7	-	1.795	2.462	No
8	10	2.041	2.462	Yes

By using Grubbs' test outliers are detected in pharmacies 1, 3, 4, and 8 for the product with a shortage in supply. The results of outliers are summarized in Table 14.

It is essential to mention that almost half of the pharmacies have outliers during the analyzed period.

## Discussion

The author tested forecasting methods by using different planning levels and outlier detection procedures. The results using the forecasting method incorporating seasonality aspect Holt–Winters Multiplicative method with outlier detection give better results, which has to be applied on the lowest planning level with summing forecasts to estimate the total volume per pharmacy chain. However, if the sales of drugs are increasing, the research results could be improved by adding safety stock to the results reached with the forecasting method application. The drugs with the increase of sales are potential to be in shortage as the method follows historical data that in certain time of period the sales could not show the upcoming increase in sales. However, this is not tested in this study but could be used as the scenario for further research.

The seasonality aspect was revised by using Google trend statistics on drug search data as recommended by Rossignol et al. (24) for spring and summer in the year 2021, and it was evident that during COVID the seasonality aspect was not a case that requires separate investigation.

After the literature review, it is identified that the number of forecast periods should be selected based on the maximum lead-time that is necessary to deliver the product to the individual pharmacy. The study focuses on a short term period but a long-term perspective could be researched in case of necessity.

In summary, as the study has some limitations and does not incorporate long-term forecasting and the safety stock for drugs with increasing sales, it does not reduce the advantages of the study, having high MAPE results compared with other papers and the incorporation of planning levels.

## Conclusions

The review of the literature shows that several methods Holt's linear and Holt-Winters no trend was not getting high attention from authors seeking to forecast the sales of drugs. The author also figured out the gap in the literature, which suggests about planning levels that a basically the case in the production literature. Due to that the author tried to show the differences in the study how the selection of planning levels impacts the forecasting results. The results of the study show that the application of Holt-Winters Multiplicative method helps to improve the forecast weekly, where one of ten products is in shortage. The paper presents the forecasting model, which is dependent on four different approaches representing the divisions of the pharmacy chain. The article presents impressive empirical research results. After the application of eight forecasting methods for the product without shortage, the third approach is proven and for the product with a shortage - the fourth approach. Both tests show that forecasting at the pharmacy level is applicable for both products. After all, forecasts performed on the pharmacy level are summed up to get the total estimates for a pharmacy chain. The best forecasting method is selected by applying Theil's U_2_ statistics, which in most cases, presented the best results for Holt-Winters Multiplicative method. The application of this method shows that Theil's U_2_ value is minimized and the application of this method could support the reduction of shortage in drug supply. On the other hand, it is quite a robust and straightforward forecasting technique. Such technique is especially evident for the product with shortage, where without replacement of data sets for shortage and recovery periods to mean value, only the naive method delivers the best forecasting results. Results prove that before the forecast for the product with shortage, the technique of missing data and outlier detection must be incorporated into the forecasting model. Overwise, it would be oversupplied or undersupplied.

## Data Availability Statement

The original contributions presented in the study are included in the article/supplementary material, further inquiries can be directed to the corresponding author.

## Author Contributions

The author confirms being the sole contributor of this work and has approved it for publication.

## Funding

Funding will be arranged at VILNIUS Gediminas technical university.

## Conflict of Interest

The author declares that the research was conducted in the absence of any commercial or financial relationships that could be construed as a potential conflict of interest.

## Publisher's Note

All claims expressed in this article are solely those of the authors and do not necessarily represent those of their affiliated organizations, or those of the publisher, the editors and the reviewers. Any product that may be evaluated in this article, or claim that may be made by its manufacturer, is not guaranteed or endorsed by the publisher.
